# Well-integrated ZnO nanorod arrays on conductive textiles by electrochemical synthesis and their physical properties

**DOI:** 10.1186/1556-276X-8-28

**Published:** 2013-01-15

**Authors:** Yeong Hwan Ko, Myung Sub Kim, Wook Park, Jae Su Yu

**Affiliations:** 1 Department of Electronics and Radio Engineering, Institute for Laser Engineering, Kyung Hee University, 1 Seocheon-dong, Giheung-gu, Yongin-si, Gyeonggi-do, 446-701, Republic of Korea

**Keywords:** ZnO nanorod arrays, Electrochemical deposition, Conductive textile

## Abstract

We reported well-integrated zinc oxide (ZnO) nanorod arrays (NRAs) on conductive textiles (CTs) and their structural and optical properties. The integrated ZnO NRAs were synthesized by cathodic electrochemical deposition on the ZnO seed layer-coated CT substrate in ultrasonic bath. The ZnO NRAs were regularly and densely grown as well as vertically aligned on the overall surface of CT substrate, in comparison with the grown ZnO NRAs without ZnO seed layer or ultrasonication. Additionally, their morphologies and sizes can be efficiently controlled by changing the external cathodic voltage between the ZnO seed-coated CT substrate and the counter electrode. At an external cathodic voltage of −2 V, the photoluminescence property of ZnO NRAs was optimized with good crystallinity and high density.

## Background

Vertically aligned one-dimensional (1D) zinc oxide (ZnO) nanostructures, such as nanorod and nanowire arrays, have attracted much interest in various application fields including field-emission devices, light-emitting diodes, piezoelectric devices, dye-sensitized solar cells, chemical sensors, and photodetectors due to their superior structural and optical properties compared to the bulk structure [1-7]. Over the past decade, there have been many efforts for controlling the structural and morphological properties of the 1D ZnO nanostructures with high density and uniformity because their size, shape, distribution, and crystallinity are closely related to the physical properties [8-10]. Furthermore, the hierarchical architectures built by the 1D ZnO nanostructures with 2D or 3D templates, which look like flowers or urchins, have potentially exhibited the improvements of device performance due to the highly extended surface area and density [11-14]. Nowadays, some vigorous attempts begin to be focused on the growth and deposition of the 1D ZnO nanostructures on various functional material substrates, for example, indium tin oxide-coated polyethylene terephthalate (i.e., ITO/PET) films, metal foils, graphenes, and cellulose fibers, thus leading to the merits of flexible and bendable feasibility with light weight and low cost [15-18].

On the other hand, the fabrication technique of conductive textiles (CTs) has been considerably developed by utilizing an electroless metallization of polymer fibers, and thus they have been used for electromagnetic interference shielding fabrics and flexible electrodes [19,20]. In addition, the CTs can be a promising candidate as substrate for integrating the 1D ZnO nanostructures by employing the electrochemical deposition (ED) method. When electrons are supplied into the conductive surface in growth solution, ZnO nanorods can be readily synthesized and controlled at a low temperature by varying the external cathodic voltage [15,21]. Therefore, the ED process with CT substrate can be a powerful and convenient fabrication method for preparing the vertically aligned 1D ZnO nanostructures on a conductive and flexible substrate. In this paper, we synthesized and controlled the integrated ZnO nanorod arrays (NRAs) on nickel (Ni)-coated PET fiber CTs by ED method with different external cathodic voltages. For more regular and dense ZnO NRAs, the CTs were coated by the ZnO seed solution, and the samples were treated by ultrasonic agitation during ED process.

## Methods

All chemicals were purchased from Sigma-Aldrich (St. Louis, MO, USA), which were of analytical grade. To synthesize the ZnO NRAs on CT substrates, we used the commercially available CT substrates which consisted of woven Ni-plated PET (i.e., Ni/PET) fibers. For preparing the working substrate, the CT substrate of 3 × 3 cm^2^ was cleaned by ethanol and deionized (DI) water in ultrasonic bath for 10 min, respectively, at room temperature. The seed solution was made by dissolving the 10 mM of zinc acetate dehydrate (Zn(CH_3_COO)_2_ 2H_2_O) in 50 ml of ethanol and by adding 1.5 wt.% of sodium dodecyl sulfate solution (CH_3_(CH_2_)_11_OSO_3_Na). After that, the CF substrates were dipped into the seed solution and pulled up slowly. To achieve good adhesion between the coated seed layer and the surface of CT, the samples were placed in the oven at 130°C for 2 h. Meanwhile, the aqueous growth solution was prepared by dissolving the 10 mM of zinc nitrate hexahydrate (Zn(NO_3_)_2_ 6H_2_O) and 10 mM of hexamethylenetetramine ((CH_2_)_6_ N_4_) in 900 ml of DI water at 74 to 76°C under magnetic stirring. For growing the ZnO NRAs via the ED process, we used a simple two-electrode system containing the working electrode (i.e., deposited sample) and counter electrode (i.e., platinum mesh) since it is convenient and cost-effective for the synthesis of metal oxides nanostructures [22,23]. For providing reliable information on the growth condition in ED process, the time-dependent applied current densities were recorded at different external cathodic voltages. In order to investigate the effect of external cathodic voltage on the growth property of ZnO NRAs, the samples were fabricated at various cathodic voltages from −1.6 to −2.8 V for 1 h. Herein, the pH value of growth solution was measured in the range of approximately 6.25 to 6.5 during the ED process. The morphologies and structural properties were observed by using a field-emission scanning electron microscope (FE-SEM; LEO SUPRA 55, Carl Zeiss, Reutlingen, Germany) and a transmission electron microscope (TEM; JEM 200CX, JEOL, Tokyo, Japan). The crystallinity and optical property were analyzed by the X-ray diffraction (XRD; M18XHF-SRA, Mac Science Ltd., Yokohama, Japan) patterns and the photoluminescence (PL; RPM2000, Accent Optical Technologies, York, UK) spectra, respectively.

## Results and discussion

**Figure 1:**
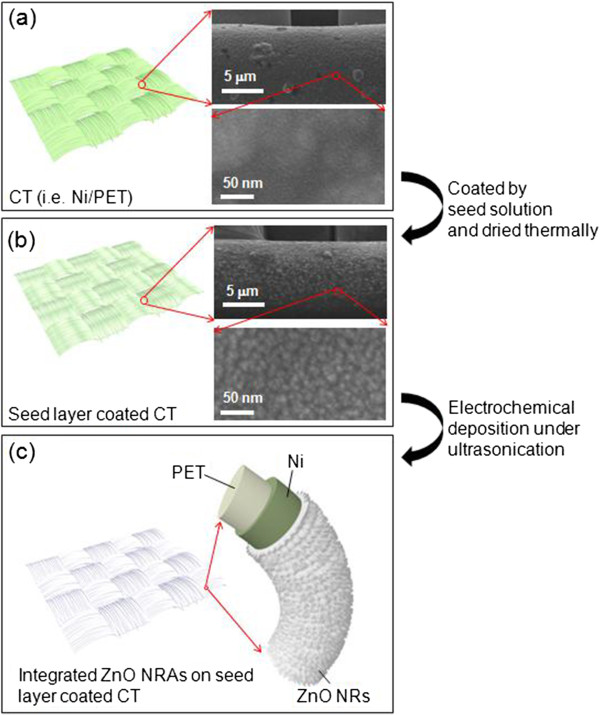
**Schematic diagram.** ED process for the ZnO NRAs on CT substrates. (**a**) The preparation of CT substrate, (**b**) the ZnO seed-coated CT substrate, and (**c**) the integrated ZnO NRAs on the seed-coated CT substrate.

Figure 1 shows the schematic diagram of ED process for the ZnO NRAs on CT substrates and their corresponding FE-SEM images including Figure 1a, the preparation of CT substrate; Figure 1b, the ZnO seed-coated CT substrate; and Figure 1c, the integrated ZnO NRAs on the seed-coated CT substrate. Here, the ED process was carried out under ultrasonic agitation. As shown in Figure 1a, the flexible Ni/PET fibers with diameters of approximately 20 μm were woven into the textile. After the CT substrate was coated by the seed solution and dried thermally, a thin ZnO seed layer was formed, as can be seen in the SEM image of Figure 1. When the seed-coated CT substrate was immersed into the growth solution and supplied by electrons, the seed layer provided ZnO crystal nuclei sites which allowed for growing the ZnO NRAs densely and vertically. As compared in the SEM images of Figure 1a,b, it can be clearly observed that the ZnO seed of approximately 5 to 20 nm was coated on the surface of Ni/PET fibers. Therefore, as shown in Figure 1c, the ZnO NRAs can be integrated into the whole surface of Ni/PET fibers after the ED process, thanks to the seed layer and ultrasonication. Typically, in ED process, the zinc hydroxide (Zn(OH)_2_) nanostructure is formed at the surface of seed layer and it is changed into the ZnO nanostructure by dehydration. As the cathodic voltage is applied externally to the working electrode, the hydroxide (OH^−^) ions are produced at the seed layer due to the reduction of precursors including the nitrate ions and hydrogen peroxides [24]. In that time, the Zn^2+^ ions are diffused into the seed layer by the Coulombic attraction under strong electric field and then combined with OH^−^ ions. Finally, the ZnO NRAs are formed and self-assembled with a preferred growth directionality of *c*-axis in wurtzite crystal structure.

**Figure 2:**
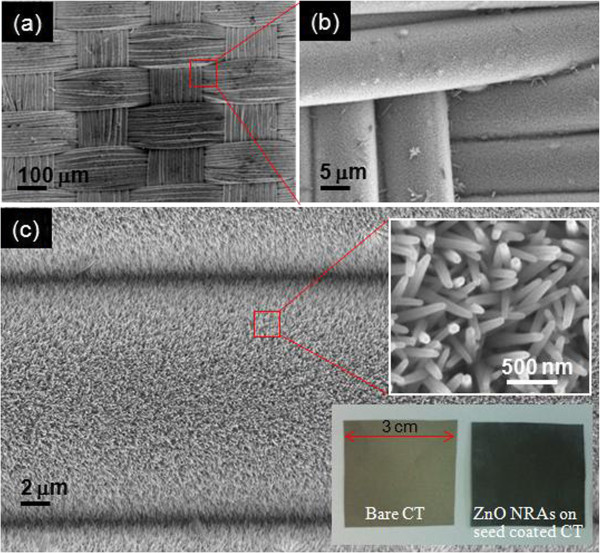
**FE-SEM micrographs.** Integrated ZnO NRAs on the seed-coated CT substrate at an external cathodic voltage of −2 V for 1 h under ultrasonic agitation. (**a**) Low magnification, (**b**) medium magnification, and (**c**) high magnification. The insets of (**c**) show the magnified FE-SEM image of the selected region and the photographs of the bare CT and the ZnO NRAs integrated CT substrate.

Figure 2 shows the SEM images of the integrated ZnO NRAs on the seed-coated CT substrate at an external cathodic voltage of −2 V for 1 h under ultrasonic agitation. The insets of Figure 2c show the magnified SEM image of the selected region and the photographs of the bare CT and the ZnO NRAs-integrated CT substrate. In the perspective view of the sample in Figure 2a, the shape of the textile was kept intact. With a closer view, as shown in Figure 2b, the ZnO NRAs were densely and clearly coated over the overall surface of Ni/PET fibers with few ZnO microrods. During the ED process, indeed, the ZnO was formed not only at the surface of seed layer, but also in the growth solution because some Zn^2+^ ions react with the remaining OH^−^ ions supported from hexamethylenetetramine. Therefore, some zinc hydroxides were created and grown into the microrods in growth solution, which were attached at the already organized ZnO NRAs on the seed layer. For this reason, the ultrasonic agitation was employed to avoid such attachments. As shown in Figure 2c, it can be clearly observed that the ZnO nanorods were aligned with varying vertical angle and integrated with the regular-sized ones. The sizes/heights of ZnO nanorods were approximately estimated to be about 65 to 80 nm/600 to 800 nm. From the photographs, the ZnO NRAs were clearly deposited on the seed-coated CT substrate. Additionally, the ZnO NRAs-integrated CT substrate became much darker compared to the bare CT substrate due to the antireflection effect, because the ZnO NRAs provide a graded effective refractive index profile between air and the CT substrate [25,26]. Therefore, the CT substrate can absorb more light from air via the ZnO NRAs due to the reduced surface reflection, thus leading to a black-colored surface like black silicon [27].

**Figure 3:**
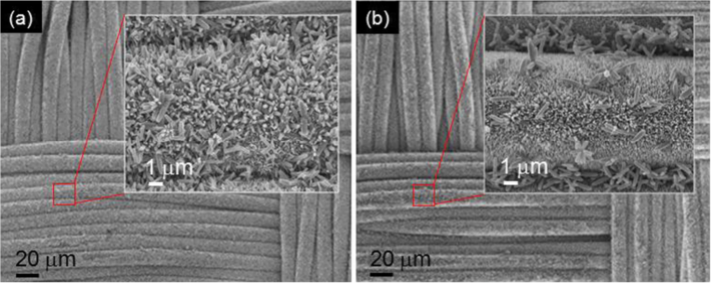
**FE-SEM micrographs.** ZnO NRAs grown on (**a**), the bare CT substrate with the ultrasonic agitation; and (**b**), the seed-coated CT substrate without the ultrasonic agitation. For comparison, the external cathodic voltage and growth time were −2 V and 1 h, respectively, as the same condition of Figure 2.

To investigate the effects of seed layer and ultrasonic agitation on the growth property, the ZnO NRAs were synthesized on bare CT substrate in ultrasonic bath (i.e., without seed layer) and were also synthesized on the seed-coated CT substrate without ultrasonic agitation. Figure 3 shows the SEM images of the ZnO NRAs grown on Figure 3a, the bare CT substrate with the ultrasonic agitation; and in Figure 3b, the seed-coated CT substrate without the ultrasonic agitation For comparison, the external cathodic voltage and growth time were −2 V and 1 h, respectively, as the same condition of Figure 2. As shown in Figure 3a, the ZnO NRAs were grown on the seedless CT substrate. In fact, it was previously understood that the ZnO NRAs could be formed with no seed layer by the ED process [28,29]. However, the size and distribution of ZnO nanorods were not regular and the vertical alignment was poor. Since the ZnO nuclei were randomly created and organized without seed layer, the ZnO nanorods were formed with different sizes and they were aligned obliquely along each growth direction. For the grown sample without the aid of ultrasonic agitation in Figure 3b, on the contrary, the ZnO NRAs were densely and vertically formed, but many microrods were attached to them. As explained in Figure 2, some zinc hydroxides were already formed in growth solution, and the microrods readily adhered to the ZnO NRAs when the ultrasonic agitation was not applied to the aqueous growth solution. Therefore, the seed layer and ultrasonic agitation are crucial to obtain the well-integrated ZnO NRAs on CT substrates.

**Figure 4:**
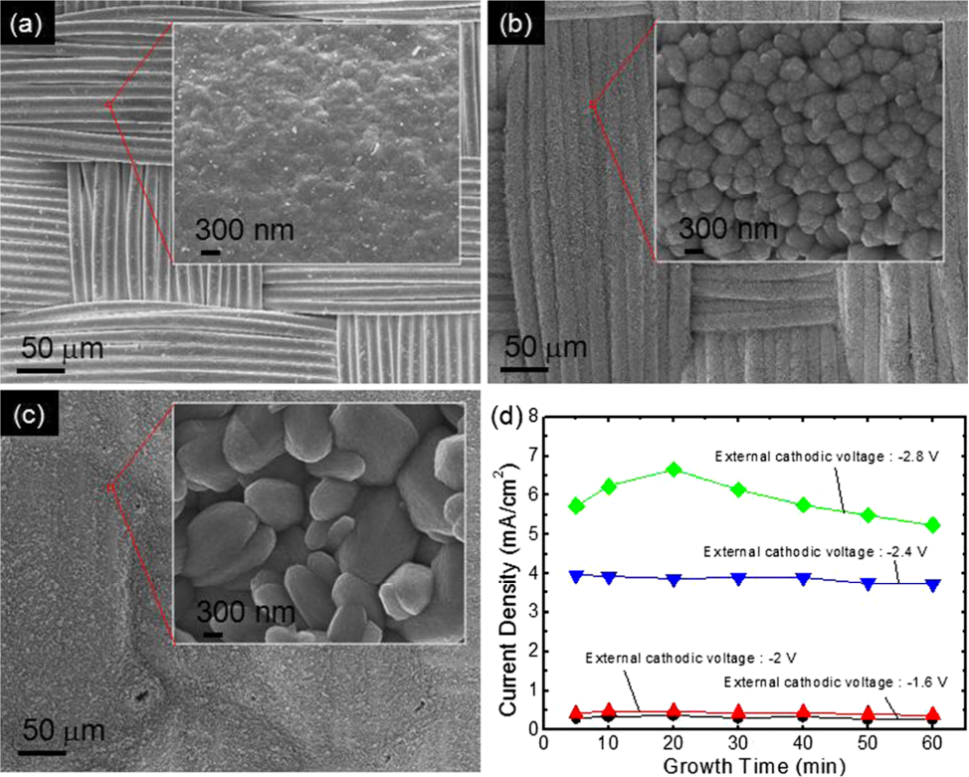
**FE-SEM micrographs and applied current densities.** Synthesized ZnO on the seed-coated CT substrate at different external cathodic voltages of (**a**) −1.6 V, (**b**) −2.4 V, and (**c**) −2.8 V for 1 h under ultrasonic agitation, and (**d**) current density as a function of growth time at different external cathodic voltages. The insets of (**a** to **c**) show the magnified SEM images of the selected region of the corresponding samples.

Figure 4 shows the SEM images for the synthesized ZnO on the seed-coated CT substrate at different external cathodic voltages of Figure 4a, −1.6 V; Figure 4b, −2.4 V; and Figure 4c, −2.8 V for 1 h under ultrasonic agitation; and Figure 4d, the current density as a function of growth time at different external cathodic voltages. The insets of Figure 4a,b,c show the magnified SEM images of the selected region of the corresponding samples. Below −1.6 V of external cathodic voltage, the ZnO NRAs could not be formed due to the insufficient electron supply under a low external cathodic voltage. In contrast, the size of ZnO was dramatically increased with increasing the external cathodic voltage to −2.4 and −2.8 V. In general, the ZnO nanorods may be grown anisotropically under ED conditions. While the Zn^2+^ ions diffuse rapidly into the polar plane, they cannot diffuse into the nonpolar plane relatively because the hexamine molecules were early attached to the ZnO pillars, thus blocking out the reaction between the Zn^2+^ and OH^−^ ions [30]. Accordingly, the ZnO nanorods are grown along the polar planes corresponding to the *c*-axis of wurtzite crystal structure. At high external cathodic voltage, however, the strong electric field causes the faster diffusion of Zn^2+^ ions which leads to the combination with the OH^−^ ions at the pillars. The size of ZnO nanorods becomes larger due to the isotropic growth. At −2.4 V, the shape of the CTs was still kept, but the boundaries between the Ni/PET fibers were somewhat not well-defined in Figure 4b. As shown in the inset, the sizes of thick ZnO microstructures were estimated to be approximately 0.5 to 1 μm and their surface looked like a porous film due to the closely packed ZnO microstructures. When the external cathodic voltage was increased to −2.8 V, the deposited ZnO was much thicker and the shape of the CTs was indistinguishable (Figure 4c). As can be seen in the inset, the sizes of thick ZnO microstructures were distributed to be approximately 2.5 to 4 μm. Figure 4d shows the measured current densities at different external cathodic voltages. During the ED process for 1 h, the current densities were observed to be about 0.25 to 0.35, 0.37 to 0.47, 3.74 to 3.97, and 5.24 to 6.67 mA/cm^2^ at the external cathodic voltages of −1.6, −2, −2.4 and −2.8 V, respectively. At low external cathodic voltages of −1.6 and −2 V, the current density was slightly changed and stabilized. But the current density somewhat fluctuated at high external cathodic voltage of −2.4 V, and it became more unstable at −2.8 V. This is probably attributed to the large variation of electrolyte at high external cathodic voltage.

**Figure 5:**
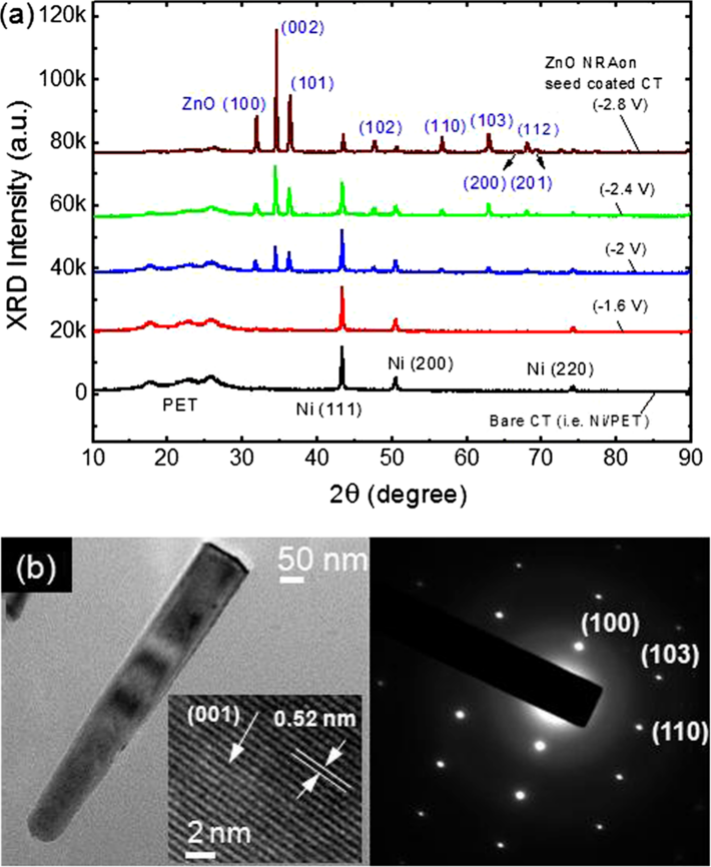
**XRD patterns and TEM images.** (**a**) Synthesized ZnO on the seed-coated CT substrate at different external cathodic voltages from −1.6 to −2.8 V for 1 h under ultrasonic agitation, and (**b**) TEM image (left) and SAED pattern (right) of the single nanorod detached from the ZnO NRAs grown at −2 V. For comparison, the XRD pattern of bare CT substrate is also given in (a). The inset of (b) shows the HR TEM image of the ZnO nanorod.

Figure 5a shows the 2θ scan XRD patterns of the synthesized ZnO on the seed-coated CT substrate at different external cathodic voltages from −1.6 to −2.8 V for 1 h under ultrasonic agitation, and Figure 5b shows the TEM image and selected area electron diffraction (SAED) pattern of the single nanorod detached from the ZnO NRAs grown at −2 V. For comparison, the XRD pattern of bare CT substrate is also given in Figure 5a. The high-resolution (HR) TEM image of the ZnO nanorod is also shown in the inset of Figure 5b. As can be seen in all XRD patterns, the PET and Ni peaks were clearly observed at the same positions. At −1.6 V, meanwhile, it was difficult to observe the ZnO XRD peaks since the ZnO was not formed as shown in Figure 4a. However, when the external cathodic voltage was increased above −2 V, the ZnO XRD peaks were clearly observed. Herein, the ZnO XRD patterns were indexed to the wurtzite structure of ZnO (JCPDS card number 89-1397). For three ZnO-deposited samples (−2, −2.4, and −2.8 V), the dominant ZnO (002) peaks were commonly observed, indicating that the ZnO was preferentially grown along the *c*-axis. As the external cathodic voltage was increased from −2 to −2.8 V, the ZnO (002) peak intensity was gradually increased and the Ni/PET peaks were decreased relatively. This may be caused by the thicker and closely packed ZnO as shown in Figure 4. To obtain a single ZnO nanorod for TEM images and SAED patterns, the ZnO NRAs integrated sample (Figure 2) was agitated in ethanol solution by ultrasonication. In Figure 5b, the single ZnO nanorod with size/height of 75/600 nm was shown, and the indexed SAED pattern confirmed that the ZnO nanorod was well crystallized with the wurtzite structure. As can be seen in the inset of Figure 5b, the lattice spacing of 0.52 nm was observed in the lattice fringes, which was also in well agreement with the d-spacing of the ZnO (002) crystal plane corresponding to 2θ = 34.4°.

**Figure 6:**
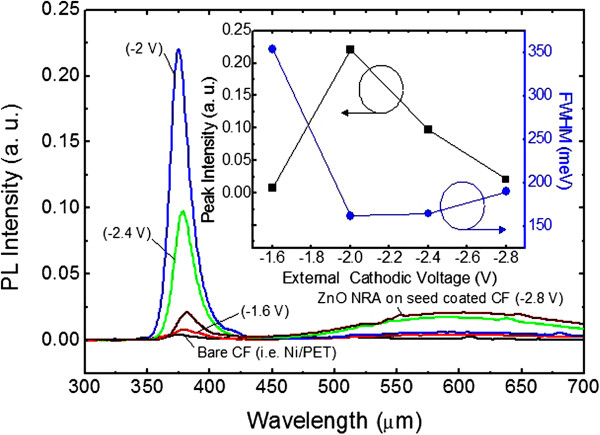
**Room-temperature PL spectra.** Bare CT substrate and the synthesized ZnO on the seed-coated CT substrate at different external cathodic voltages from −1.6 to −2.8 V for 1 h under ultrasonic agitation. The inset shows the PL peak intensity and FWHM of the synthesized ZnO as a function of external cathodic voltage.

Figure 6 shows the room-temperature PL spectra of the bare CT substrate and the synthesized ZnO on the seed-coated CT substrate at different external cathodic voltages from −1.6 to −2.8 V for 1 h under ultrasonic agitation. The inset shows the PL peak intensity and full width at half maximum (FWHM) of the synthesized ZnO as a function of external cathodic voltage. Here, the PL emission was detected with an excitation at 266 nm using an Nd-YAG laser source. For the bare CT substrate, there was no PL emission peak due to the absence of the ZnO. Similarly, for the rarely synthesized ZnO on the seed-coated CT substrate under a low external cathodic voltage of −1.6 V, a very weak PL emission peak was observed in the ultraviolet (UV) wavelength region. However, for the ZnO-deposited samples with external cathodic voltages of −2, −2.4, and −2.8 V, the narrow PL emission peaks were observed at wavelengths of 374.3, 377.8, and 380.2 nm, respectively. These PL emissions were attributed to the near band edge (NBE) transition and radial recombination in the direct bandgap of the deposited ZnO. Particularly, the PL intensity of UV emission was largely increased at −2 V (i.e., integrated ZnO NRAs on the seed-coated CT substrate). As shown in the inset, the PL intensity of UV emission at −2 V was increased by 10.5 times compared to that at −2.8 V and its FWHM was also minimized to 162 meV. This enhancement was caused mainly by the size and density of ZnO NRAs. As the size of ZnO nanorods is decreased and their surface area is increased, the incident photon-to-electron conversion efficiency and PL property can be improved [31]. However, the PL intensity of UV emission was degraded with increasing the external cathodic voltage above −2 V because the synthesized material further contains the Zn according to Pourbaix diagram for Zn^2+^ in aqueous solution [32]. Consequently, the well-integrated ZnO NRAs on the CT substrate could be fabricated by the ED process with the aid of ultrasonic agitation under a proper external cathodic voltage.

## Conclusions

The ZnO NRAs were successfully integrated on the CT substrate (i.e., woven by Ni/PET fibers) by the ED process using the seed layer and ultrasonic agitation under a proper external cathodic voltage of −2 V for 1 h. The sizes/heights of ZnO NRAs were distributed to be approximately 65 to 80 nm/600 to 800 nm, and they could be clearly coated over the whole surface of the CT substrate with the seed layer and ultrasonic agitation. In a comparative investigation, it is clearly observed that the seed layer and ultrasonic agitation played key roles in providing a uniform organization of the ZnO NRAs with good nuclei sites as well as removing the adhesive ZnO microrods. Additionally, the well-integrated ZnO NRAs exhibited a narrow and strong PL NBE emission with good crystallinity. This optimal ED process for the well-integrated ZnO NRAs on CT substrates can be an essential growth technique for producing flexible and wearable functional materials in ZnO-based optoelectronic and electrochemical devices.

## Competing interest

The authors declare that they have no competing interests.

## Authors’ contributions

YHK designed and optimized the synthesis of the ZnO NRAs on CF substrate by the ED process. MSK assisted the technical support for measurements (FE-SEM, TEM, XRD, and PL). WP analyzed the experimental data. JSY developed the conceptual framework, supervised the whole work, and drafted the manuscript. All authors read and approved the final manuscript.
